# Independent Chromatin Binding of ARGONAUTE4 and SPT5L/KTF1 Mediates Transcriptional Gene Silencing

**DOI:** 10.1371/journal.pgen.1002120

**Published:** 2011-06-09

**Authors:** M. Jordan Rowley, Maria I. Avrutsky, Christopher J. Sifuentes, Ligia Pereira, Andrzej T. Wierzbicki

**Affiliations:** Department of Molecular, Cellular, and Developmental Biology, University of Michigan, Ann Arbor, Michigan, United States of America; University of Geneva, Switzerland

## Abstract

Eukaryotic genomes contain significant amounts of transposons and repetitive DNA elements, which, if transcribed, can be detrimental to the organism. Expression of these elements is suppressed by establishment of repressive chromatin modifications. In *Arabidopsis thaliana*, they are silenced by the siRNA–mediated transcriptional gene silencing pathway where long non-coding RNAs (lncRNAs) produced by RNA Polymerase V (Pol V) guide ARGONAUTE4 (AGO4) to chromatin and attract enzymes that establish repressive chromatin modifications. It is unknown how chromatin modifying enzymes are recruited to chromatin. We show through chromatin immunoprecipitation (ChIP) that SPT5L/KTF1, a silencing factor and a homolog of SPT5 elongation factors, binds chromatin at loci subject to transcriptional silencing. Chromatin binding of SPT5L/KTF1 occurs downstream of RNA Polymerase V, but independently from the presence of 24-nt siRNA. We also show that SPT5L/KTF1 and AGO4 are recruited to chromatin in parallel and independently of each other. As shown using methylation-sensitive restriction enzymes, binding of both AGO4 and SPT5L/KTF1 is required for DNA methylation and repressive histone modifications of several loci. We propose that the coordinate binding of SPT5L and AGO4 creates a platform for direct or indirect recruitment of chromatin modifying enzymes.

## Author Summary

Transposons and other repetitive elements occupy vast areas of the eukaryotic genomes. They pose a threat to genome integrity but at the same time regulate expression of many genes and have been proposed to be a major factor contributing to genome evolution. One of the processes responsible for controlling activity of transposons and other repetitive elements is transcriptional gene silencing. This process uses small interfering RNA and long non-coding RNA to recruit enzymes that establish repressive chromatin modifications. Several proteins have been identified to be needed for siRNA–mediated transcriptional silencing in *Arabidopsis thaliana*, however for many of them their position in the silencing pathway is unknown. One of those proteins is SPT5L/KTF1, a homolog of an elongation factor associated with RNA Polymerase II. Here we establish the position of SPT5L in the silencing pathway and propose the molecular mechanism of its function. This gives further knowledge of the mechanism of transcriptional gene silencing and is important to understand how transposons are controlled.

## Introduction

Eukaryotic genomes contain significant amounts of transposons and other repetitive DNA elements, which usually remain transcriptionally inactive. Efficient silencing of transposon transcription is essential for preventing their mobility and for maintaining genome integrity [1]. Transposon silencing has also been hypothesized to regulate expression of genes that contain transposable elements in their promoters and to facilitate the evolution of genomes [2].

Transposons are silenced at both transcriptional and post-transcriptional levels by mechanisms that involve small interfering RNAs (siRNAs) [3]. These 20–25-nt RNA molecules are generated by the RNase III enzyme Dicer and provide sequence specificity for effector complexes mediating RNA cleavage and/or the establishment of chromatin modifications that silence transcriptional activity [3]. In *Arabidopsis thaliana*, single-stranded RNA precursors for siRNA biogenesis are produced by RNA Polymerase II (Pol II) or RNA Polymerase IV (Pol IV), while the second strand is synthesized by RDR2 (RNA-Dependent RNA Polymerase 2). DCL3 (Dicer-like 3) cleaves double-stranded RNA into siRNAs that are then incorporated into ARGONAUTE4 (AGO4) [4], [5]. This mechanism seems to be similar in maize where homologs of RDR2 and Pol IV have been shown to be involved in transcriptional gene silencing [6]–[8].

Recognition of target loci by AGO4-siRNA complexes requires sequence identity between siRNAs and the genomic loci. These loci, however, are often actively transcribed, and it is not clear if siRNAs base-pair interact with DNA or nascent RNA transcripts [3], [9]. The latter possibility is well supported in *Schizosaccharomyces pombe* where loci subject to siRNA-mediated transcriptional silencing are actively transcribed by RNA Polymerase II [10]–[12]. The central role of nascent transcripts in recognition of siRNA targets in *S. pombe* was observed by the ability of Argonaute proteins to cleave RNA. This ability is required for the establishment of repressive chromatin modifications [13]. Moreover, tethering Argonaute and siRNA-containing RITS (RNA-induced initiation of transcriptional gene silencing) complex to nascent transcripts is sufficient for the initiation of repressive chromatin modifications and transcriptional silencing [14].

This mechanism may be similar in *Arabidopsis* where transcriptional silencing requires a specialized RNA Polymerase complex known as RNA Polymerase V (Pol V) [15]–[17]. Pol V produces non-coding transcripts in otherwise silent chromatin, and its activity is required for the establishment and maintenance of repressive chromatin modifications [18]. Pol V-produced non-coding transcripts physically interact with AGO4 and recruit siRNA-AGO4 complexes to their targets [19]. Additionally, transcriptional silencing of several loci needs AGO4 slicer activity [20], suggesting that in plants siRNAs may recognize their targets by base-pair interactions with Pol V transcripts [19].

RNA Polymerases and AGO4 are assisted in their functions by several other known protein components of the plant silencing system, all of which are required for efficient establishment and maintenance of transcriptional silencing [5]. One of them is SPT5L (Suppressor of Ty insertion 5 - like; also known as SPT5-like or KTF1), a homolog of SPT5 Pol II-associated elongation factor. It was shown to contain a domain rich in WG/GW repeats that facilitate physical interaction with AGO4 [21]–[23]. Because SPT5L interacts with RNA but is not required for the accumulation of Pol V-dependent transcripts, it was hypothesized to work downstream of Pol V and recruit AGO4 to Pol V-transcribed loci [22], [24].

Despite the recent progress in understanding the mechanisms of transcriptional gene silencing, it is not known how siRNAs work with Pol V transcripts, AGO4 and other proteins to recruit chromatin modifying enzymes to their target loci in chromatin. It is unknown how chromatin-bound AGO4 recruits enzymes that establish repressive chromatin modifications. It is unknown if other protein components of the silencing system help AGO4 recruit chromatin modifying enzymes. It is also unknown in what order proteins involved in silencing are recruited to chromatin. Here we try to resolve the mechanism of siRNA-mediated recruitment of chromatin modifying enzymes to chromatin and the function of SPT5L in this process. We show that SPT5L physically interacts with chromatin and that SPT5L works downstream of Pol V but does not require 24-nt siRNA. SPT5L and AGO4 are recruited to chromatin in parallel and at least partially independently of each other and both are needed for DNA methylation and repressive histone modifications at several loci. We propose that the coordinate binding of SPT5L and AGO4 creates a platform for direct or indirect recruitment of chromatin modifying enzymes.

## Results

### SPT5L interacts with chromatin

**Figure 1:**
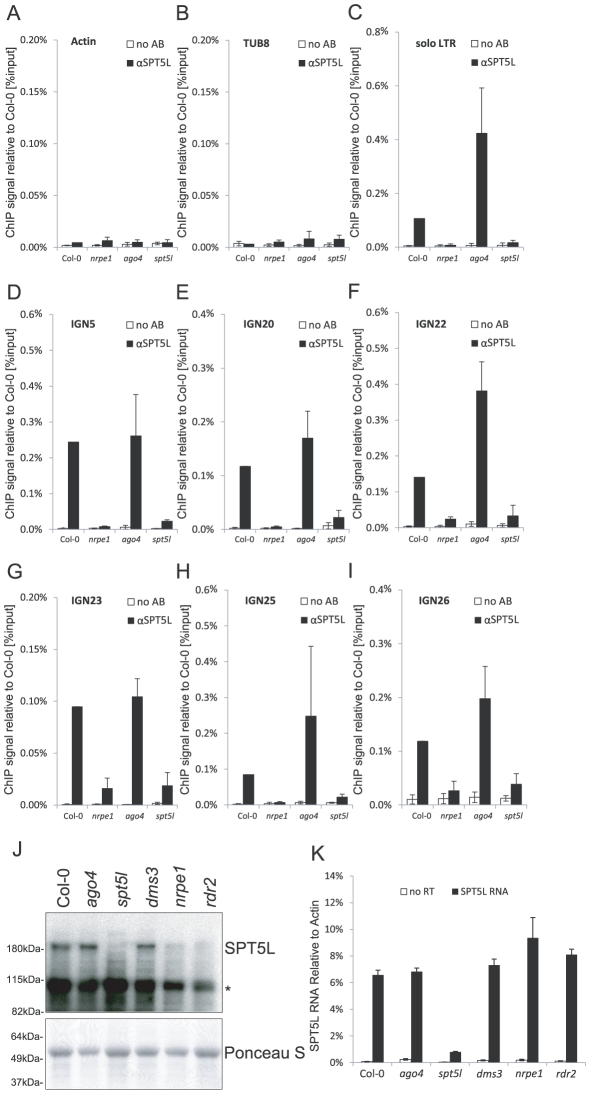
SPT5L interacts with chromatin in a Pol V-dependent and AGO4-independent manner. (A–I) ChIP data showing SPT5L binding to chromatin in Col-0 wild type, *nrpe1*, *ago4* and *spt5l* mutants at loci transcribed by Pol V and silenced by siRNA-mediated transcriptional silencing: *solo LTR*(C), *IGN5* (D), *IGN20* (E), *IGN22* (F), *IGN23* (G), *IGN25* (H) and *IGN26* (I). Two loci transcribed by Pol II are shown as controls: *Actin 2* (A) and *Tubulin 8* (B). No antibody controls (white bars) provide background level for ChIP samples (black bars). Bars represent mean value of ChIP signals normalized to Col-0 wild type. Error bars are standard deviations of three independent biological replicates. (J) Immunoblot detection of SPT5L in whole-cell protein extracts from Col-0 wild type, *ago4*, *spt5l*, *dms3*, *nrpe1* and *rdr2* mutants. Ponceau S staining of the membrane is a loading control. Asterisk denotes nonspecific bands. (K) Real time RT-PCR detection of *SPT5L* RNA in Col-0 wild type, *ago4*, *spt5l*, *dms3*, *nrpe1* and *rdr2* mutants. Bars represent average *SPT5L* mRNA accumulation relative to *Actin 2* from three biological replicates. Error bars represent standard deviation.

The interaction of SPT5L with AGO4 [21], [22] suggested that like AGO4 [19], SPT5L may bind loci targeted by siRNA-mediated transcriptional gene silencing. We first used chromatin immunoprecipitation (ChIP) with anti-SPT5L antibody to test if SPT5L binds chromatin. Subsequent real-time PCR demonstrated recovery of *IGN5* and *solo LTR* DNA from Col-0 wild type at much higher levels than from *spt5l* mutant which represents the background level (Figure 1C, 1D). This shows that SPT5L physically interacts with *IGN5* and *solo LTR* loci which are known to be transcribed by Pol V and silenced by the siRNA-mediated transcriptional gene silencing pathway [18], [19], [25], [26]. There was, however, no enrichment on the control *Actin 2* and *Tubulin 8* (*TUB 8*) loci (Figure 1A, 1B), which are transcribed by Pol II and not occupied by components of the silencing pathway [18], [27]. This suggests that SPT5L is present at the loci undergoing transcriptional silencing and that its function in silencing is most likely direct.

Interaction of SPT5L with chromatin was also demonstrated at *IGN20*, *IGN22*, *IGN23*, *IGN25* and *IGN26* (Figure 1E–1I), which have been identified in a genome-wide screen of Pol V occupancy (A. Wierzbicki, R. Lister, B. Gregory, J. Ecker and C. Pikaard, unpublished data), suggesting that SPT5L binding may be a general feature of Pol V-transcribed loci.

### SPT5L works downstream of Pol V

**Figure 2:**
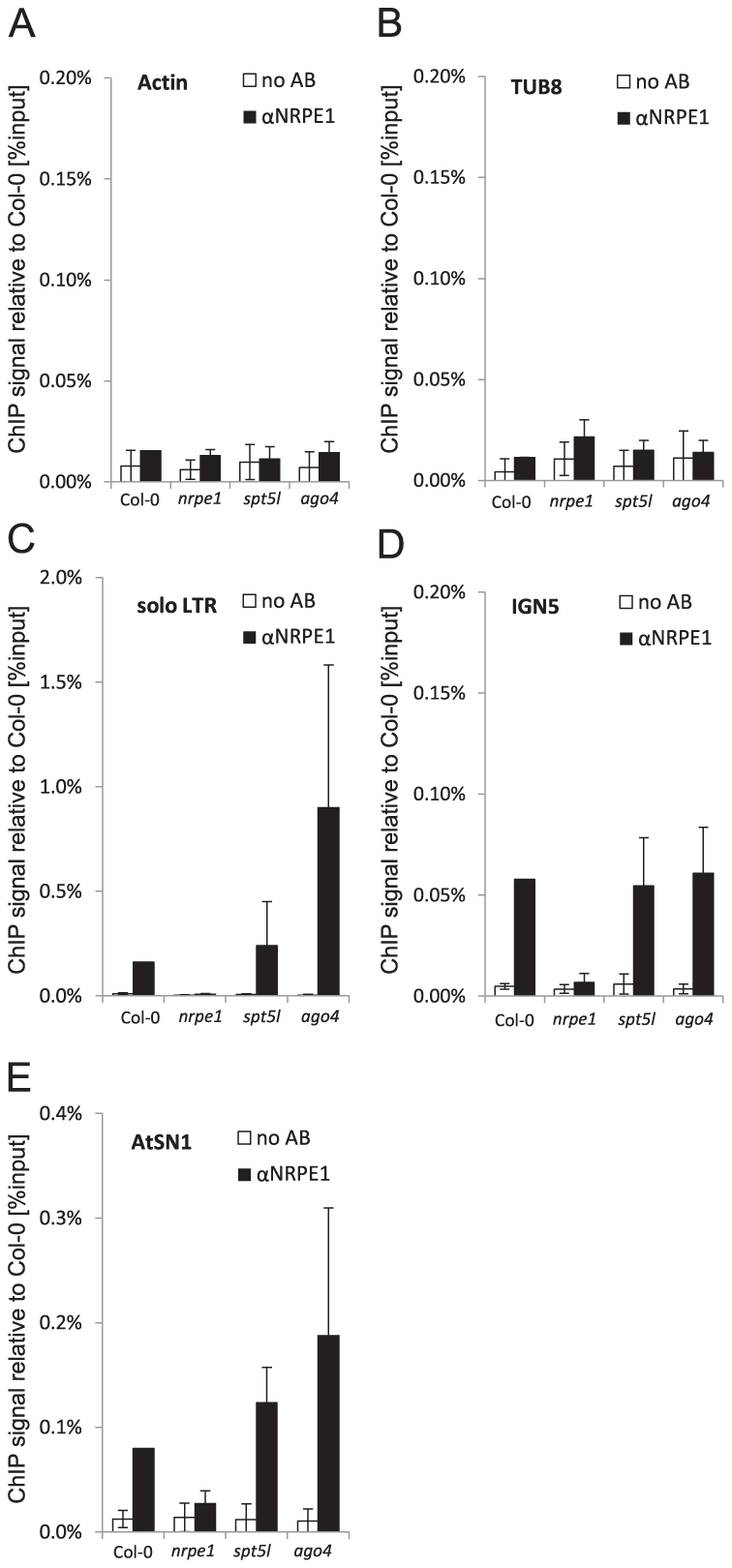
SPT5L and AGO4 are not required for Pol V binding to chromatin. (A–E) Pol V occupancy of *Actin 2* (A) and *Tubulin 8* (B) control loci, *solo LTR* (C), *IGN5* (D) and *AtSN1* (E) assayed by ChIP in Col-0 wild type, *nrpe1*, *spt5l* and *ago4*. No antibody controls (white bars) provide background level for ChIP samples (black bars). Bars represent mean value of ChIP signals normalized to Col-0 wild type. Error bars are standard deviations of three independent biological replicates.

SPT5L interacts with chromatin (Figure 1C–1I) as well as with AGO4, Pol V complex and Pol V transcripts [21]–[23]. SPT5L is also not required for the accumulation of Pol V-dependent transcripts at *IGN5*, *IGN6* or *AtSN1* [22]. This suggests that SPT5L should work downstream of Pol V. To test this prediction we assayed Pol V binding to chromatin by ChIP with antibody against NRPE1, the largest subunit of Pol V. Subsequent real-time PCR demonstrated recovery of DNA from Col-0 wild type at much higher level than from the *nrpe1* mutant at *IGN5*, *solo LTR* and *AtSN1* loci but not at *Actin 2* or *Tubulin 8* loci (Figure 2A–2E) demonstrating that Pol V binds chromatin at *IGN5*, *solo LTR* and *AtSN1* loci. DNA recovery from *spt5l* mutant was comparable to Col-0 wild type (Figure 2A–2E) showing that SPT5L is not needed for Pol V binding to chromatin. Interestingly, Pol V binding to chromatin was reproducibly increased at *solo LTR* locus in *ago4* mutant (Figure 2C), indicating that AGO4 may inhibit Pol V binding to chromatin possibly by affecting initiation and/or elongation of Pol V transcription. We conclude that SPT5L does not work upstream of Pol V in siRNA-mediated transcriptional gene silencing pathway.

Because both Pol V and SPT5L are required for DNA methylation at several silenced loci [21]–[23], SPT5L may be functionally dependent on Pol V and/or Pol V transcription. To test this possibility we performed western blot with anti-SPT5L antibody in *nrpe1* mutant background. Accumulation of SPT5L was strongly reduced in the *nrpe1* mutant (Figure 1J). To test if *nrpe1* mutation affects accumulation of *SPT5L* mRNA or SPT5L protein stability, we assayed *SPT5L* RNA using real time RT-PCR. Accumulation of *SPT5L* RNA was not reduced in the *nrpe1* mutant (Figure 1K) indicating that Pol V is needed for SPT5L protein stability. This behavior of SPT5L in *nrpe1* mutant is reminiscent of reduced AGO4 protein stability in mutants that reduce siRNA production [28]. Interestingly, we observed a slight increase in *SPT5L* RNA level in the *nrpe1* mutant which may be explained by the presence of an AtMU10 transposon in *SPT5L* coding region. Overall, these results suggest that SPT5L is functionally dependent on Pol V.

We further tested the functional relationship between Pol V and SPT5L by performing ChIP with anti-SPT5L antibody in *nrpe1* mutant background. Consistent with the reduced stability of SPT5L in *nrpe1* (Figure 1J), DNA recovery from Pol V-transcribed loci was reduced to the level observed in the *spt5l* mutant (Figure 1C–1I). This result may be explained by the overall reduction in the amount of SPT5L. However, a similar reduction in the SPT5L protein accumulation in *rdr2* mutant (Figure 1J) did not affect SPT5L binding to chromatin (see below). This suggests that *nrpe1* may affect the ChIP signal not only by destabilizing SPT5L, but also by affecting its ability to bind chromatin. Because SPT5L does not work upstream of Pol V and is functionally dependent on Pol V, we conclude that SPT5L works downstream of Pol V and/or Pol V transcription and may be recruited to chromatin by Pol V.

### SPT5L binds chromatin independently of AGO4

The recruitment of SPT5L to chromatin by Pol V (Figure 1, Figure 2) is consistent with the interaction of SPT5L with Pol V transcripts and AGO4 [21], [22]. There are at least two explanations of SPT5L function in the establishment of siRNA-mediated transcriptional gene silencing. SPT5L may be recruited by Pol V and then help recruit AGO4-siRNA complexes. Alternatively, AGO4-siRNA may recognize target loci and then recruit SPT5L which further recruits chromatin modifying enzymes. To test the latter possibility we performed ChIP with αSPT5L antibody in the *ago4* mutant. DNA recovery of all tested Pol V-transcribed loci was comparable from Col-0 wild type and the *ago4* mutant (Figure 1C–1I). This shows that binding of SPT5L to chromatin was not affected in the *ago4* mutant, and suggests that SPT5L is not recruited to its target loci by AGO4-siRNA complexes. We conclude that SPT5L does not work downstream of AGO4 in the siRNA-mediated transcriptional gene silencing pathway.

### AGO4 binds chromatin partially independently of SPT5L

**Figure 3:**
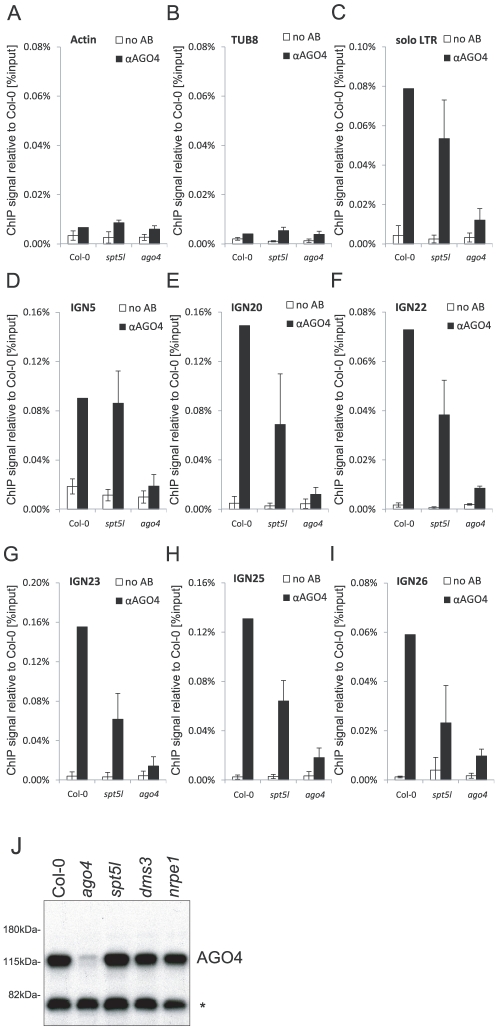
AGO4 can bind chromatin independently of SPT5L. (A–I) ChIP data showing AGO4 binding to chromatin in Col-0 wild type, *spt5l* and *ago4* mutants at *Actin 2* (A) and *Tubulin 8* (B) control loci, *solo LTR* (C), *IGN5* (D), *IGN20* (E), *IGN22* (F), *IGN23* (G), *IGN25* (H) and *IGN26* (I). No antibody controls (white bars) provide background level for ChIP samples (black bars). Bars represent mean value of ChIP signals normalized to Col-0 wild type. Error bars are standard deviations of three independent biological replicates. (J) Immunoblot detection of AGO4 in whole-cell protein extracts from Col-0 wild type, *ago4*, *spt5l*, *dms3* and *nrpe1* mutants using anti-AGO4 antibody. Asterisk denotes a nonspecific band. Ponceau S staining of the membrane shown in Figure 1J is a loading control.

Having concluded that SPT5L does not work downstream of AGO4, we tested the alternative hypothesis that SPT5L may work upstream of AGO4 by binding Pol V and/or Pol V transcripts and recruiting AGO4 to chromatin. To test this possibility we performed ChIP with anti-AGO4 antibody. As demonstrated by real-time PCR we recovered DNA from wild type plants above the background level observed in the *ago4* mutant at *IGN5* and *solo LTR* (Figure 3C, 3D) as well as at *IGN20*, *IGN22*, *IGN23*, *IGN25* and *IGN26* loci (Figure 3E–3I). This indicates that AGO4 binds chromatin at all tested Pol V-transcribed loci. In the *spt5l* mutant total accumulation of AGO4 protein was not affected (Figure 3J). At all assayed Pol V-transcribed loci AGO4 binding to chromatin in the *spt5l* mutant was reproducibly above the background level observed in the *ago4* mutant indicating that AGO4 is able to bind chromatin in the absence of SPT5L (Figure 3C–3I). Interestingly, we observed that the intensity of AGO4 binding to chromatin is slightly reduced in the *spt5l* mutant at *solo LTR*, *IGN20*, *IGN22*, *IGN23*, *IGN25* and *IGN26* (Figure 3C–3I). This indicates that although SPT5L is not required for AGO4 recruitment to chromatin, it enhances AGO4 chromatin binding. Alternatively, most loci may be occupied by two pools of AGO4. One being SPT5L-dependent and other recruited to chromatin independently of SPT5L.

We conclude that SPT5L is not required for recruitment of a pool of AGO4 to specific loci in chromatin and therefore does not work upstream of AGO4 in the siRNA-mediated transcriptional gene silencing pathway. Since SPT5L also does not work downstream of AGO4, they are most likely recruited in parallel and at least partially independently of each other.

### SPT5L binds chromatin independently of 24-nt siRNA

**Figure 4:**
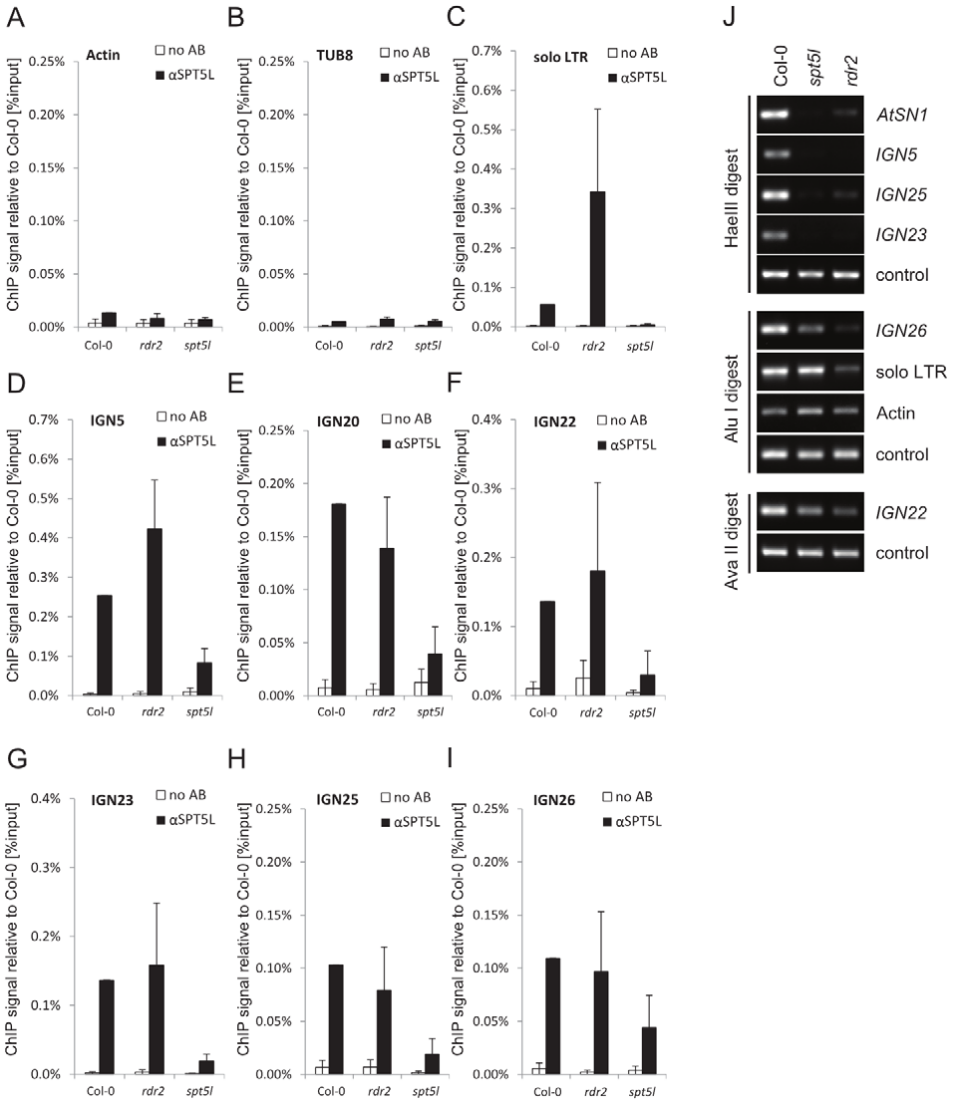
SPT5L interacts with chromatin in an siRNA–independent manner. (A–I) ChIP data showing SPT5L binding to chromatin in Col-0 wild type, *rdr2* and *spt5l* mutants at loci transcribed by Pol V and silenced by siRNA-mediated transcriptional silencing: *solo LTR*(C), *IGN5* (D), *IGN20* (E), *IGN22* (F), *IGN23* (G), *IGN25* (H) and *IGN26* (I). Two loci transcribed by Pol II are shown as controls: *Actin 2* (A) and *Tubulin 8* (B). No antibody controls (white bars) provide background level for ChIP samples (black bars). Bars represent mean value of ChIP signals normalized to Col-0 wild type. Error bars are standard deviations of three independent biological replicates. (J) DNA methylation analysis of *AtSN1*, *IGN5*, *IGN23* and *IGN25* performed by digestion with *Hae*III restriction endonuclease, *IGN26* and *solo LTR* performed by digestion with *Alu*I restriction endonuclease and *IGN22* performed by digestion with *Ava*II restriction endonuclease. Digested genomic DNA was amplified by PCR. Sequences lacking *Hae*III (*Actin 2*), *Alu*I (*IGN5*) or *Ava*II (*Actin 2*) were used as loading controls.

The parallel and independent recruitment of SPT5L and AGO4 to chromatin suggests that they are both guided by the interactions with Pol V complex and/or Pol V transcripts. To test if SPT5L is also guided by siRNA we used ChIP to assay SPT5L binding to chromatin in *rdr2*, a mutant in an RNA-dependent RNA polymerase responsible for production of the majority of 24-nt siRNA [29]. The *rdr2* mutation reduced the stability of SPT5L protein (Figure 1J, 1K) but did not cause reduction in DNA recovery of the tested loci after ChIP (Figure 4C–4I). This suggests that although RDR2 increases the amount of SPT5L protein, the chromatin-bound fraction of SPT5L is not affected by the *rdr2* mutation. This also suggests the presence of siRNA-dependent pool of SPT5L that does not physically interact with assayed Pol V-transcribed loci.

These results demonstrate that binding of SPT5L to chromatin is not affected in the *rdr2* mutant and suggest that RDR2-dependent siRNA is not required for SPT5L binding to chromatin. In contrast, RDR2 is necessary for proper establishment of DNA methylation at *AtSN1*, *IGN5*, *IGN25*, *IGN23*, *IGN26*, *solo LTR* and *IGN22* (Figure 4J); demonstrating that all assayed loci are in fact targets of the siRNA-mediated transcriptional gene silencing pathway. We conclude that SPT5L is recruited to chromatin in a manner independent of 24-nt siRNA.

### Both AGO4 and SPT5L are needed for repressive chromatin modifications

**Figure 5:**
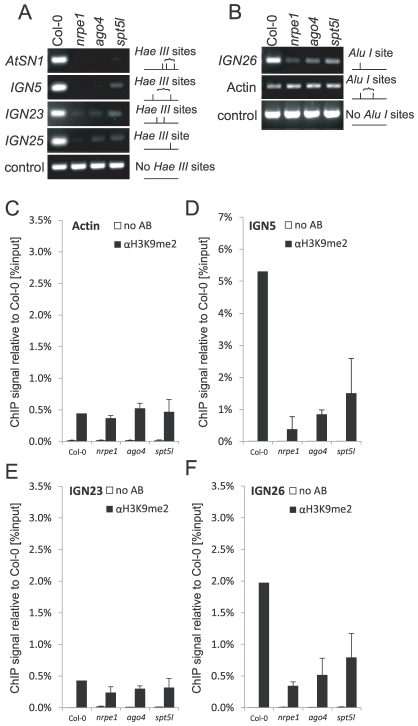
Both SPT5L and AGO4 are required for silencing at certain loci. (A) DNA methylation analysis of *AtSN1*, *IGN5*, *IGN23* and *IGN25* performed by digestion with *Hae*III restriction endonuclease. Digested genomic DNA was amplified by PCR. Sequence lacking *Hae*III (*Actin 2*) sites was used as a loading control. (B) DNA methylation analysis of *IGN26* performed by digestion with *Alu*I restriction endonuclease. Digested genomic DNA was amplified by PCR. Sequence lacking *Alu*I (*IGN5*) sites was used as a loading control. (C–F) Analysis of H3K9me2 at *actin 2* (C), *IGN5* (D), *IGN23* (E) and *IGN26* (F) loci performed by ChIP with anti-H3K9me2 antibody in Col-0 wild type, *nrpe1*, *ago4* and *spt5l*. No antibody controls (white bars) provide background level for ChIP samples (black bars). Bars represent mean value of ChIP signals normalized to Col-0 wild type. Error bars are standard deviations of three independent biological replicates.

Parallel and at least partially independent recruitment of SPT5L and AGO4 by Pol V suggests that at Pol V-transcribed loci none of them is sufficient for the establishment and maintenance of silent chromatin modifications. To further test this possibility we assayed several Pol V-transcribed loci for DNA methylation side-by-side in *nrpe1*, *ago4* and *spt5l* mutants using DNA methylation-sensitive restriction endonucleases. Methylation of cytosines in *Hae*III, *Alu*I or *Ava*II restriction sites blocks the enzymes from cutting and allows amplification of the genomic region by PCR. However, unmethylated sites are cleaved and PCR amplification fails. All three enzymes recognize asymmetric (CHH) methylation at tested loci. Consistently with previous reports, DNA methylation was strongly reduced at *AtSN1* locus in both *ago4* [19], [24], [30] and *spt5l* mutants [21]–[23] and at *IGN5* locus in *ago4* mutant [19] (Figure 5A). DNA methylation was also reduced at *IGN5* locus in *spt5l* mutant and at *IGN23*, *IGN25* and *IGN26* loci in both *ago4* and *spt5l* mutants (Figure 5A, 5B). Importantly, in all these cases reduction of DNA methylation was comparable in *ago4* and *spt5l* mutants (Figure 5A, 5B) suggesting that neither AGO4 nor SPT5L is sufficient for the establishment of asymmetric DNA methylation at Pol V-transcribed loci.

We also tested the effect of *nrpe1*, *ago4* and *spt5l* mutations on dimethylation of lysine 9 of histone H3 (H3K9me2). At *IGN5* and *IGN26* loci, H3K9me2 was reduced in all three mutants (Figure 5D, 5F) showing that both AGO4 and SPT5L are required not only for the establishment and/or maintenance of DNA methylation but also H3K9me2. We conclude that at least at a subset of loci SPT5L and AGO4 work together to recruit repressive chromatin modifications. We propose that it is the coordinate action of SPT5L and AGO4 that directly or indirectly recruits *de novo* DNA methyltransferase DRM2 and H3K9 methyltransferases.

### SPT5L contributes to repressive chromatin modifications in a locus-specific manner

**Figure 6:**
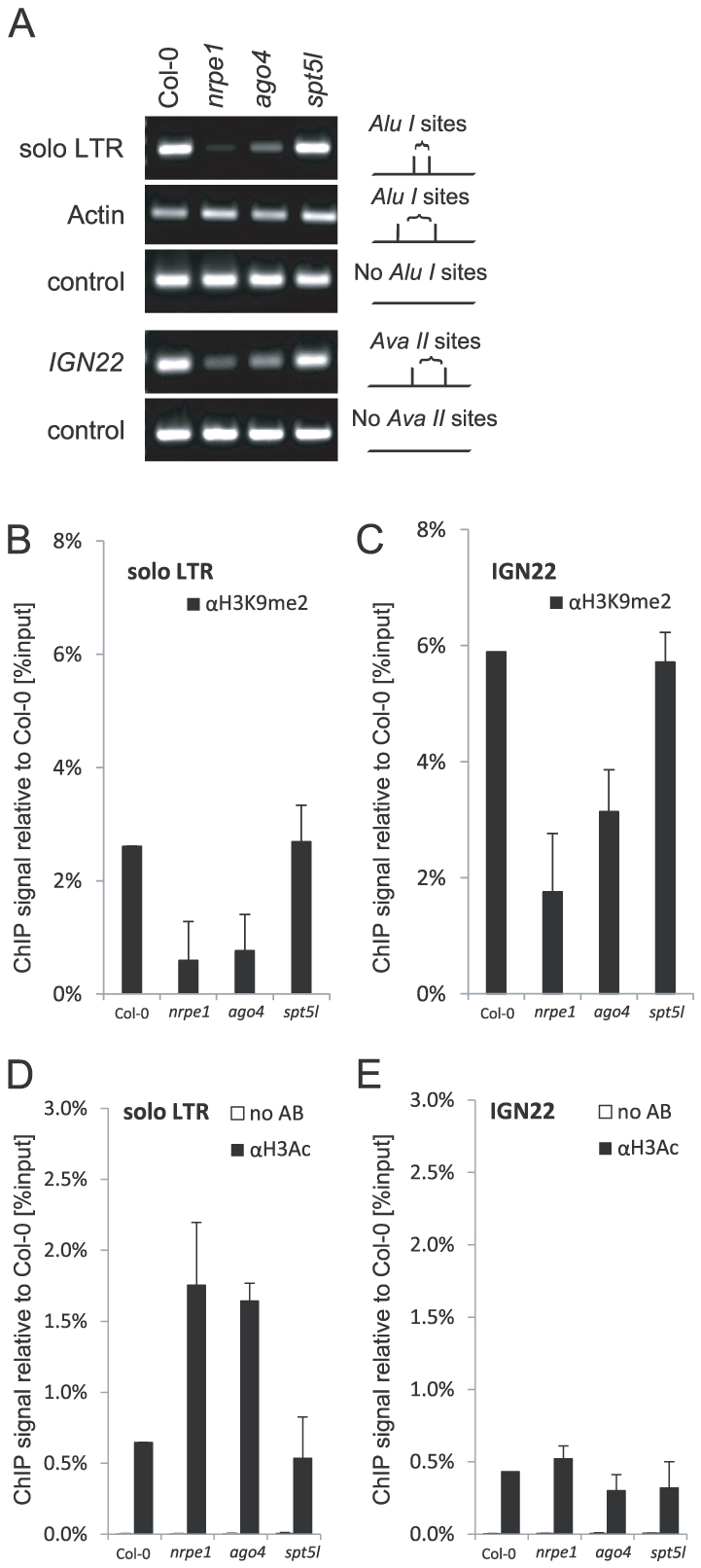
Locus-specific effects of SPT5L on silencing. (A) DNA methylation analysis of *solo LTR* performed by digestion with *Alu*I restriction endonuclease and *IGN22* performed by digestion with *Ava*II restriction endonuclease. Digested genomic DNA was amplified by PCR. Sequences lacking *Alu*I (*IGN5*) or *Ava*II (*Actin 2*) sites were used as loading controls. (B–C) ChIP analysis of H3K9me2 at *solo LTR* (B) and *IGN22* (C) in Col-0 wild type, *nrpe1*, *ago4* and *spt5l*. Corresponding no antibody controls are shown in panels D and E. Bars represent mean value of ChIP signals normalized to Col-0 wild type. Error bars are standard deviations of three independent biological replicates. (D–E) ChIP analysis of H3Ac at *solo LTR* (D) and *IGN 22* (E) in Col-0 wild type, *nrpe1*, *ago4* and *spt5l*. No antibody controls (white bars) provide background level for ChIP samples (black bars). Bars represent mean value of ChIP signals normalized to Col-0 wild type. Error bars are standard deviations of three independent biological replicates.

While *AtSN1*, *IGN5*, *IGN23*, *IGN25* and *IGN26* loci require both AGO4 and SPT5L for repressive chromatin modifications (Figure 5), *soloLTR* has been shown to be methylated independently of SPT5L [21], [23]. We confirm this result and further show that *solo LTR* and *IGN22* which, like other Pol V-transcribed loci, are methylated in a Pol V and AGO4-dependent manner (Figure 6A) did not show reduction of DNA methylation on *Alu*I or *Ava*II sites in the *spt5l* mutant (Figure 6A). This suggests that there is some significant locus specificity in SPT5L contributions to DNA methylation. Furthermore, H3K9me2 was reduced at both *soloLTR* and *IGN22* in *nrpe1* and *ago4* mutants but not in the *spt5l* mutant (Figure 6B, 6C). Also acetylation of histone H3 (H3Ac) at *solo LTR* was increased in *nrpe1* and *ago4* but not in *spt5l* (Figure 6D). This demonstrates that the locus-specific function of SPT5L affects not only DNA methylation but also H3K9me2 and H3Ac.

The requirement of SPT5L for repressive chromatin modifications (Figure 5, Figure 6) does not correlate with the extent of partial SPT5L-dependency of AGO4 binding to chromatin (Figure 3). It suggests that the pool of AGO4 that is bound to chromatin in an SPT5L-dependent manner is not required for silencing. This is consistent with our interpretation that AGO4 and SPT5L are recruited to chromatin in parallel and independently of each other.

## Discussion

### Order of events in siRNA–mediated silencing

Our findings establish the order of events leading to siRNA-mediated establishment of transcriptional silencing. This process is initiated by recognition of silencing targets and production of two classes of non-coding RNA. The first class is siRNA which is produced from double-stranded RDR2 products by DCL3 and becomes incorporated into AGO4 and possibly also AGO6 and AGO9 [4], [5], [24]. The second class is long non-coding RNA produced by Pol V and/or Pol II [18], [26]. Pol V transcription is initiated independently of siRNA and Pol V transcripts most likely are not precursors for siRNA biogenesis [18], [31]. Pol V recruitment to chromatin and transcription requires the presence of DMS3, DRD1 and RDM1, which either help initiate Pol V transcription or assist elongation of Pol V transcripts [18], [19], [32].

**Figure 7:**
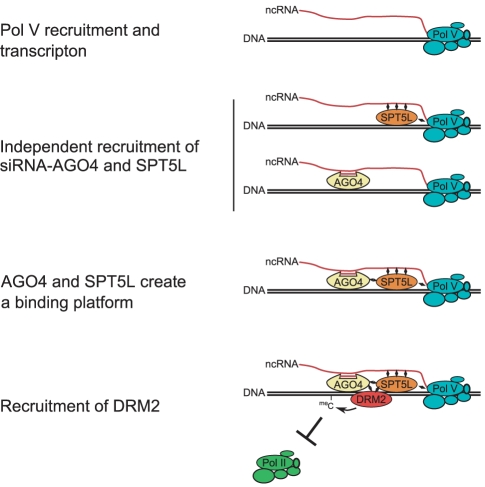
Model of SPT5L involvement in the recruitment of chromatin modifying enzymes. Pol V produces intergenic non-coding transcripts which are the binding points for SPT5L and AGO4-siRNA complex. Both AGO4 and SPT5L may interact with both Pol V transcripts and Pol V complex itself. SPT5L and AGO4 are recruited to Pol V transcripts in parallel and independently of each other. When both AGO4 and SPT5L are present they create a binding platform for direct or indirect recruitment of DRM2 *de novo* DNA methyltransferase and other chromatin modifying enzymes. Establishment of chromatin modifications represses Pol II transcription.

Pol V transcription is followed by association of two RNA-binding proteins with chromatin (Figure 7). First is AGO4 which is recruited to chromatin by Pol V transcripts and uses the incorporated siRNA to provide sequence-specificity of silencing [19]. The second is SPT5L (Figure 1, Figure 2), which is recruited to chromatin by an unknown mechanism, possibly involving interactions between SPT5L and Pol V complex and/or with Pol V transcripts [22], [23]. SPT5L binds chromatin independently of 24-nt siRNA (Figure 4) and is likely a general factor associated with transcribing Pol V and its transcripts [21]–[23]. Since SPT5L binds chromatin in the absence of AGO4 (Figure 1), and the functional pool of AGO4 is able to bind chromatin in the absence of SPT5L (Figure 3), we concluded that they are recruited to chromatin in parallel and independently of each other. Both AGO4 and SPT5L are required for the establishment and/or maintenance of DNA methylation and repressive histone modifications at the majority of tested loci (Figure 5). This suggests that both are needed for the recruitment of enzymes establishing repressive chromatin modifications.

### Mechanism recruiting chromatin modifying enzymes

Because AGO4 and SPT5L bind chromatin independently of each other, and, at the majority of tested loci both are required for establishment and maintenance of silencing, we propose that AGO4 and SPT5L create a binding platform for the recruitment of chromatin modifying proteins. One possibility is that both weakly interact with a downstream protein but the interaction becomes strong enough to recruit chromatin modifying enzymes only when both are present. Alternatively, AGO4 may be a sole interacting partner of downstream proteins but SPT5L, which has a C-terminal domain rich in WG/GW motifs, interacts with AGO4 and alters its conformation to facilitate the recruitment of chromatin modifying enzymes.

Our results show that there are loci where DNA methylation is established in a Pol V, AGO4 and SPT5L-dependent manner (Figure 5A), but these loci have an overall low level of H3K9me2 and no change in the histone modifications in tested mutants (*IGN23* in Figure 5E). It suggests that the *de novo* DNA methyltransferase DRM2 is likely the chromatin modifying enzyme directly recruited by the AGO4-SPT5L platform. It is also possible that DRM2 may be recruited indirectly by another protein that binds the AGO4-SPT5L platform.

### Assembly of the silencing complexes

Binding of AGO4 and SPT5L to chromatin is mediated by multiple protein-protein and protein-RNA interactions. These interactions may mediate recruitment of proteins to specific genomic regions and/or stabilize binding after recruitment by an independent mechanism.

SPT5L binding to chromatin occurs downstream of Pol V and is most likely mediated by protein-RNA interaction between SPT5L and Pol V transcripts [22]. Like canonical SPT5, SPT5L may also form a heterodimer with SPT4 [33]. Alternatively, SPT5L may be recruited to chromatin by protein-protein interaction with Pol V complex as suggested by identification of SPT5L in Pol V holoenzyme [23] and interactions between yeast SPT5 as well as bacterial homolog of SPT5, nusG, with RNA polymerases [34], [35]. It is also possible that SPT5L is recruited to chromatin by interacting with both Pol V transcripts and Pol V complex. All these mechanisms explain the AGO4-independent binding of SPT5L to Pol V-transcribed loci.

Interaction with Pol V transcripts seems to be the major factor recruiting AGO4-siRNA to chromatin [19]. AGO4 also interacts with WG/GW-rich C-terminal domains of Pol V and SPT5L [21], [22], [36]. Because Argonautes contain only one WG/GW binding pocket [37] these interactions may be employed sequentially. First, they help recruit AGO4 to chromatin by interaction with Pol V and then they stabilize the binding of AGO4 to chromatin on its target loci by interaction with SPT5L. It is consistent with our observation that AGO4 binding to chromatin is slightly reduced in the *spt5l* mutant (Figure 3).

### Locus-specific regulation of silencing

We show that SPT5L contributes to regulation of siRNA-mediated transcriptional silencing in a highly locus-specific manner. This is demonstrated by the observation that two of the tested loci require Pol V and AGO4 but not SPT5L for establishment and/or maintenance of repressive chromatin modifications (Figure 6). It could be explained by presence of the canonical SPT5 at a subset of silenced loci. However, both loci are occupied by SPT5L in wild type plants (Figure 1) suggesting that SPT5L is in fact involved in their silencing. Only when SPT5L is mutated, the canonical SPT5 is able to compensate the deficiency at these particular loci. Alternatively, it is possible that the observed locus-specificity of SPT5L is caused by the presence of both Pol V and Pol II at a subset of loci [26]. Pol II-bound canonical SPT5 may be able to compensate the lack of Pol V-bound SPT5L. The mechanism deciding locus specificity of the SPT5L function remains unknown.

Our results also suggest the presence of two pools of AGO4: SPT5L-dependent and SPT5L-independent. Because both pools are detectable at loci that are silenced in a SPT5L-independent manner, the SPT5L-dependent pool of AGO4 is likely not required for silencing. It may be recruited independently of siRNA by direct interaction with SPT5L and may have some other, yet unknown and locus-specific functions.

## Materials and Methods

### Plant lines and antibodies

*Arabiodopsis thaliana nrpe1* (*nrpd1b-11*), *dms3-4*, and *ago4-1* introgressed into Col-0 background were described previously [19], [38]. *rdr2-1* mutant was obtained from J. Carrington. *spt5l-1* (*rdm3-3*; SALK_001254) mutant line, affinity-purified anti-SPT5L (anti-KTF1), affinity-purified anti-Pol V (anti-NRPE1) and affinity-purified anti-AGO4 antibodies were described previously [19], [22], [39]. Mouse monoclonal anti-H3K9me2 antibody (cat. #ab1220) was obtained from Abcam, rabbit polyclonal anti-H3Ac antibody (cat. #06-599) was obtained from Millipore.

### Chromatin immunoprecipitation

ChIP was performed essentially as described [18], [19]. Detailed ChIP protocol is included in the Text S1. ChIP samples were amplified in triplicate in Applied Biosystems 7500 real time PCR machine and obtained data were analyzed using comparative C_T_ method relative to inputs [40]. All ChIP experiments were performed in three independent biological replicates. Results from every biological replicate were normalized to Col-0 wild type and normalized data were used to obtain averages and standard deviations that show fold difference between analyzed strains. Normalized data were subsequently multiplied by average ChIP signal level of Col-0 wild type. This way data are corrected for variability in overall signal strength between independent experiments, the unit is %input and presented data reflect the relative signal strength observed at particular loci. Standard deviations for Col-0 wild type are not available because Col-0 wild type was used to normalize data.

### DNA and RNA analysis

For DNA methylation analysis genomic DNA was extracted from above-ground tissue of 2-week old plants using DNeasy Plant Mini Kit (Qiagen). 100 ng of genomic DNA was digested with 10u of *Hae*III, *Alu*I or *Ava*II restriction enzymes (NEB) for 20 min. After heat-inactivation of the enzyme DNA was amplified using 0.75u Platinum Taq (Invitrogen).

Total RNA was extracted from 2-week old plants using RNeasy Plant Mini Kit (Qiagen) and amplified using SuperScript III Platinum SYBR Green One-Step qRT-PCR Kit (Invitrogen) in Applied Biosystems 7500 real time PCR machine.

Oligonucleotide primers used in this study are shown in Table S1.

## Supporting Information

Table S1 Loci assayed in this study, their accession numbers and oligonucleotide primers.
(PDF)

Text S1 Detailed Chromatin Immunoprecipitation (ChIP) protocol.
(PDF)
