# Supplementary material for: Independent Chromatin Binding of ARGONAUTE4 and SPT5L/KTF1 Mediates Transcriptional Gene Silencing
Source: PLoS Genet. 2011 Jun 9;7(6):e1002120. doi: 10.1371/journal.pgen.1002120 (PMC3111484; doi:10.1371/journal.pgen.1002120)
Supplement: Table S1 — Loci assayed in this study, their accession numbers and oligonucleotide primers. (PDF) [file pgen.1002120.s001.pdf]

Table S1

Locus	TAIR Annotation	Primer Sequence	Method	Reference
Actin	AT3G18780	GAGAGATTCAGATGCCCAGAAGTC	real-time PCR	Wierzbicki et al. 2008 [18]
		TGGATTCCAGCAGCTTCCA		
		CGAGCAGGAGATGGAAACCTCAAA	PCR	Wierzbicki et al. 2008 [18]
		AAGAATGGAACCACCGATCCAGACA		
TUB8	AT5G23860	GCTTACTAATCAAAGATGCGAGA	real-time PCR	Numa et al. 2010 [27]
		CTTGGTATCTTCCCGTCGAA		
AtSN1	AT3TE63860	CCAGAAATTCATCTTCTTTGGAAAAG	real-time PCR	Wierzbicki et al. 2008 [18]
		GCCCAGTGGTAAATCTCTCAGATAGA		
		ACCAACGTGCTGTTGGCCCAGTGGTAAATC	PCR	Wierzbicki et al. 2008 [18]
		AAAATAAGTGGTGGTTGTACAAGC		
solo LTR	AT5TE35950	GGATAGAGATGAATGATGGATAATGACA	real-time PCR	Wierzbicki et al. 2008 [18]
		TTATTTTGATCAGTGTTATAAACCGGATA		
		ATAAAACTCGAAACAAGAGTTTTCTTATTGCTTTC	PCR	Wierzbicki et al. 2008 [18]
		TAATGGTATTATTTTGATCAGTGTTATAAACCGGA		
IGN5	Between AT4TE10770 & AT4TE10775	AAGCCCAAACCATACACTAATAATCTAAT	real-time PCR	Wierzbicki et al. 2008 [18]
		CCGAATAACAGCAAGTCCTTTTAATA		
		TCCCGAGAAGAGTAGAACAAATGCTAAAA	PCR	Wierzbicki et al. 2008 [18]
		CTGAGGTATTCCATAGCCCCTGATCC		
IGN20	Between AT4G00400 & AT4G00413	GCGGTGGCTCGAGTCAAAA	real-time PCR	-
		CCTTCCTTTGTGTCGAATTAGTCCTA		
		TGTTAGCCAAAACCGACAAGAACC	PCR	-
		TTTGTCCTCGATTTTGTTCCTTCCT		
IGN22	AT4G01530	CGGGTCCTTGGACTCCTGAT	real-time PCR	-
		TCGTGACCGGAATAATTAAATGG		
		CAAAAATATTCACCCGCTACAAACAAAAA	PCR	-
		TCTTCCATTTGTGGGGCATGGT		
IGN23	AT4TE12070	GCCATTAGTTTTAGATGGACTGCAA	real-time PCR	-
		GGGCGAACCTGGAGAAAGTT		
		ACTGAAAATTGTAAACAAAGAAACGGCACTACA	PCR	-
		GATCGGTCCATAAACTTGTTGGGTTT		
IGN25	AT4TE22865	TCAAACCAAACCCCGAACTT	real-time PCR	-
		ATGCCAGAGCCTGGTGCTA		
		CTTCTTATCGTGTTACATTGAGAACTCTTTCC	PCR	-
		ATTCGTGTGGGCTTGGCCTCTT		
IGN26	Between AT4G11485 & AT4G11490	TTCCTGGCCGTTGATTGGT	real-time PCR	-
		CGTGACATTAGAAGCTCTACGAGAA		
		CTCTTTCAGTGCGACAGCCTCAT	PCR	-
		CGGCCAGGAAACCCTAACTTCC		
SPT5L mRNA	AT5G04290	TTCGTCTGCTGGTGGTTGTGCT	real-time PCR	-
		CCCGGTTTGTCATTGGTTTCTTTCT		
