# Supplementary material for: Independent Chromatin Binding of ARGONAUTE4 and SPT5L/KTF1 Mediates Transcriptional Gene Silencing
Source: PLoS Genet. 2011 Jun 9;7(6):e1002120. doi: 10.1371/journal.pgen.1002120 (PMC3111484; doi:10.1371/journal.pgen.1002120)
Supplement: Text S1 — Detailed Chromatin Immunoprecipitation (ChIP) protocol. (PDF) [file pgen.1002120.s002.pdf]

Supporting Information S1

Chromatin immunoprecipitation protocol

Three grams of above-ground tissue of 2-week old plants was crosslinked with 0.5% formaldehyde for 10 min by vacuum infiltration, followed by addition of glycine to 80 mM. Plants were rinsed with water, frozen in liquid nitrogen, ground into powder using a mortar and pestle, suspended in 25 ml of Honda Buffer (20 mM HEPES-KOH pH 7.4, 0.44 M sucrose, 1.25% ficoll, 2.5% Dextran T40, 10 mM $MgCl_2$, 0.5% Triton X-100, 5 mM DTT, 1 mM PMSF, 1% plant protease inhibitors (Sigma)), filtered through two layers of Miracloth and centrifuged at 2000 x g for 15 min. Nuclear pellets were washed three times with 1ml of Honda buffer, resuspended in Nuclei Lysis Buffer (50 mM Tris-HCl pH 8.0, 10 mM EDTA, 1% SDS, 1 mM PMSF, 1% Plant Protease Inhibitors) and DNA was fragmented to the average size of 350-500 bp by 8 to 10 pulses of sonication each 10 seconds long with 1 minute pauses in between pulses using Fisher Scientific 100 Sonic Dismembrator at power setting 1. After centrifugation at 16000 x g for 10 min., the supernatant was diluted 10-fold with 1.1% Triton X-100, 1.2 mM EDTA, 16.7 mM Tris-HCl pH 8.0, 167 mM NaCl. 25 µl of Protein A Agarose/Salmon Sperm DNA (Millipore) or Dynabeads Protein A (Invitrogen) and the appropriate antibody was added. Samples were then incubated for 8h or overnight at 4ºC on a rotating mixer. Bead-antibody complexes were washed 5 times, 5 min each, with binding/washing buffer (150 mM NaCl, 20 mM Tris-HCl pH 8.0, 2 mM EDTA, 1% Triton X-100, 0.1% SDS, 1 mM PMSF) and twice for 5 min each with 10 mM Tris-HCl pH 8.0, 1 mM EDTA. 100µl of 10% (w/v) Chelex (Bio Rad) resin, in water, was then added to the beads and crosslinking was reversed at 99 ºC for 10 min. Samples were digested with 20 µg of proteinase K (Invitrogen) for 1-2 h at 43-60 ºC followed by heat-inactivation at 95 ºC for 10 min. Alternatively, elution was performed twice with 50 µl RIP elution buffer (100 mM Tris-HCl pH 8.0, 10 mM EDTA, 1% SDS) for 20 min at 65 ºC. Samples were digested with 20 µg of proteinase K (Invitrogen) for 6h - overnight at 60 ºC. An equal volume of phenol/chloroform/isoamyl alcohol pH 6.7 25:24:1 was added to extract DNA, followed by addition of an equal volume of chloroform/isoamyl alcohol 24:1 and subsequent precipitation by addition of 2 volumes 100% EtOH, 0.1 volume 3 M Sodium Acetate and 4ul Glycoblue (Ambion). Precipitated samples were washed once with 70% EtOH and resuspended in 100ul water or TE.
